# The unlimited potential of the great pond snail, *Lymnaea stagnalis*

**DOI:** 10.7554/eLife.56962

**Published:** 2020-06-16

**Authors:** István Fodor, Ahmed AA Hussein, Paul R Benjamin, Joris M Koene, Zsolt Pirger

**Affiliations:** 1 NAP Adaptive Neuroethology, Department of Experimental Zoology, Balaton Limnological Institute, Centre for Ecological ResearchTihanyHungary; 2 Department of Ecological Sciences, Faculty of Sciences, Vrije UniversiteitAmsterdamNetherlands; 3 Sussex Neuroscience, School of Life Sciences, University of SussexBrightonUnited Kingdom; 4 Section of Animal Ecology, Department of Ecological Science, Faculty of Earth and Life Sciences, Vrije Universiteit AmsterdamAmsterdamNetherlands; eLifeUnited Kingdom; eLifeUnited Kingdom

**Keywords:** pond snail, *Lymnaea stagnalis*, natural history, neuroscience, exotoxicology, evolution, Other

## Abstract

Only a limited number of animal species lend themselves to becoming model organisms in multiple biological disciplines: one of these is the great pond snail, *Lymnaea stagnalis*. Extensively used since the 1970s to study fundamental mechanisms in neurobiology, the value of this freshwater snail has been also recognised in fields as diverse as host–parasite interactions, ecotoxicology, evolution, genome editing and 'omics', and human disease modelling. While there is knowledge about the natural history of this species, what is currently lacking is an integration of findings from the laboratory and the field. With this in mind, this article aims to summarise the applicability of *L. stagnalis* and points out that this multipurpose model organism is an excellent, contemporary choice for addressing a large range of different biological questions, problems and phenomena.

## Introduction

In ancient Greece, over 2,400 years ago, it was already recognised that by studying animals we could learn much about ourselves. Over the centuries since then, it has become clearer that some species are highly suitable in the fields of medical, basic and applied biological research (Ericsson et al., 2013). However, when considered carefully, there is perhaps only a limited set of animal species that are versatile enough to lend themselves to become model organisms in multiple biological disciplines (Frézal and Félix, 2015; Hilgers and Schwarzer, 2019; Markow, 2015; Phifer-Rixey and Nachman, 2015).

In the second half of the 20^th^ century, one booming line of research has focused on molluscs. Neuroscientists such as the Nobel Prize winners Alan Hodgkin, Andrew Huxley and Eric Kandel recognised these animals’ potential as models for understanding basic neurobiological processes (Hodgkin and Huxley, 1952; Kupfermann and Kandel, 1969; Wachtel and Kandel, 1967). One particularly well-suited mollusc for this type of research is the freshwater pond snail, *Lymnaea stagnalis*, which has been used extensively since the 1970s to study the functioning of the nervous system from molecular signalling to behaviour.

The value of *L. stagnalis* also has been recognised in a wide range of applied biological fields. These include the study of host–parasite interactions, ecotoxicology, evolution, developmental biology, genome editing, 'omics' and human disease modelling. This extensive suitability stems from the most obvious advantages of *L. stagnalis*: its well-known anatomy, development (both of the embryonic and post-embryonic processes), and reproduction biology; its well-characterised central and peripheral nervous and neuroendocrine systems from key molecules to behavioural processes; and its readily accessible and mostly large neurons. There is also a growing body of available sequence data with an impending annotated genome and the option to use new technical approaches such as genome editing. Taking all of the above into consideration, these advantages simplify the study of different scientific topics integrated from the molecular to the population level.

This article is a tribute to over 50 years of research with *L. stagnalis* that has resulted in a considerable contribution to the understanding of general biological processes. Here, we present the essential background information on the natural history of this freshwater snail. We also provide an overview of the ground-breaking and recent information on different research fields using *L. stagnalis* (and snails in general). Our aim is to showcase *L. stagnalis* as a contemporary choice for addressing a wide range of biological questions, problems and phenomena, to inspire more researchers to use this invertebrate as a model organism, and to highlight how findings from the laboratory and the field could be better integrated.

## Natural history of *L. stagnalis*

**Figure 1.**
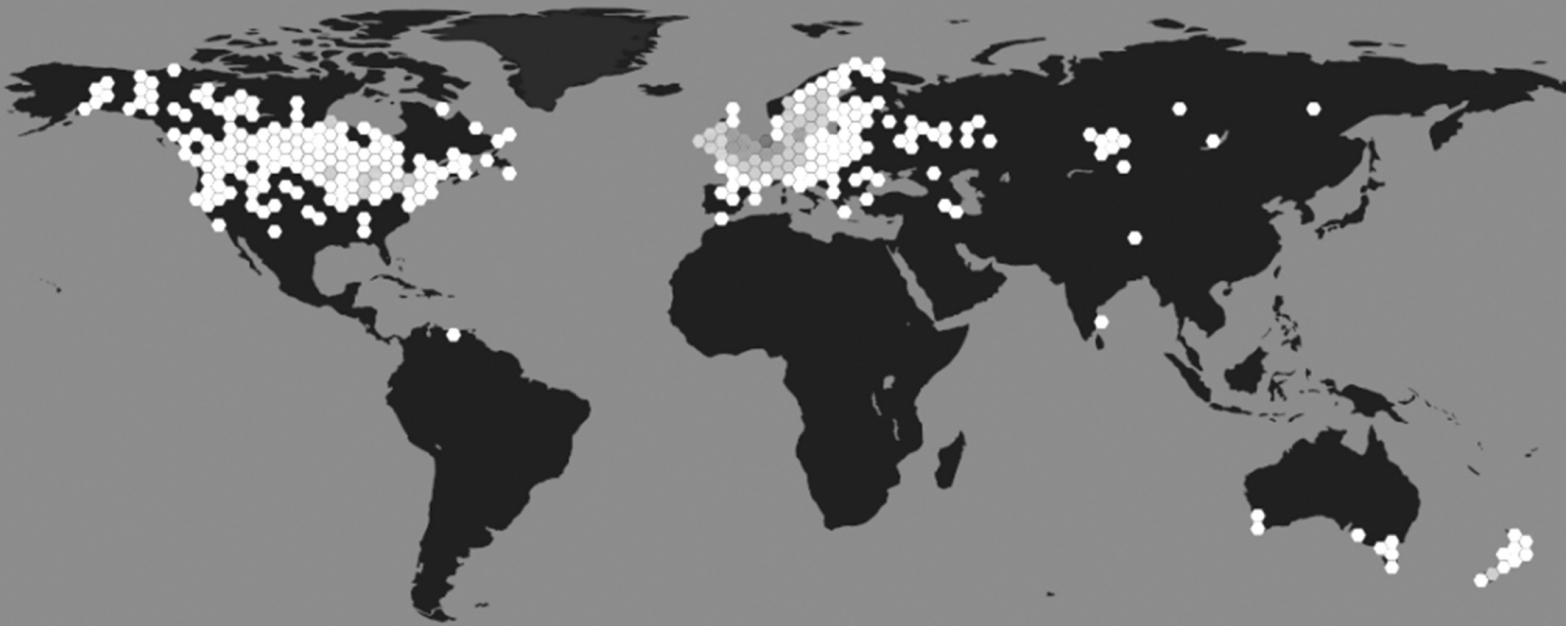
Geographical distribution of *L. stagnalis*. Places where this species of snail has been reported to occur (hexagons), shaded based on population density (white indicates low density and dark grey indicates high density; source data from GBIF Secretariat, 2019).

Initially described by Linnaeus in 1758 as *Helix stagnalis*, the species now known as *L. stagnalis* is generally referred to as the great pond snail (Panpulmonata; Hygrophila; Lymnaeidae). It is found throughout Northern America, Europe, and parts of Asia and Australia (Atli and Grosell, 2016; Zhang et al., 2018a; Figure 1). The snails inhabit stagnant and slowly running shallow waters rich in vegetation and are mainly herbivores, preferring algae, water plants and detritus (Lance et al., 2006). They are active all year round (even when there is a layer of ice on the water) but typically reproduce from spring to late autumn (Nakadera et al., 2015). They do not have a clear day-night rhythm, but display sleep-like behaviour (Stephenson and Lewis, 2011) and are more likely to lay eggs during daytime (Ter Maat et al., 2012). They are light to dark brown in colour and relatively large for pond snail species, with their spiral shells reaching lengths of up to 55 mm (Benjamin, 2008). In highly oxygenated water, they absorb oxygen directly across their body wall; but when dissolved oxygen levels drop, they switch to breathing via a lung accessed by a respiratory orifice called the pneumostome (Lukowiak et al., 1996).

*L. stagnalis* serves as the intermediate host for parasites including flatworms responsible for diseases such as fascioliasis (liver fluke and river rot) and cercarial dermatitis (swimmer’s itch) in humans (Adema et al., 1994; Davison and Blaxter, 2005; Ferté et al., 2005; Núñez et al., 1994; Skála et al., 2020). Laboratory and field studies showed that penetration of a parasite into a snail will initiate a chronic infection in which the parasite alters snail neurophysiology, metabolism, immunity, growth and reproduction (Kryukova et al., 2014; Langeloh and Seppälä, 2018; Vorontsova et al., 2019). These studies have also investigated how selection acts on immune defence traits (Langeloh et al., 2017). Investigations of the natural history of *L. stagnalis*, which focus on host-parasite associations, aid the development of novel control measures that reduce snail-mediated parasitic transmissions. Primary predators of juveniles and adults include leeches, crayfish and fish, some of which snails can detect via chemicals that the predators emit (Dalesman and Lukowiak, 2012).

**Figure 2.**
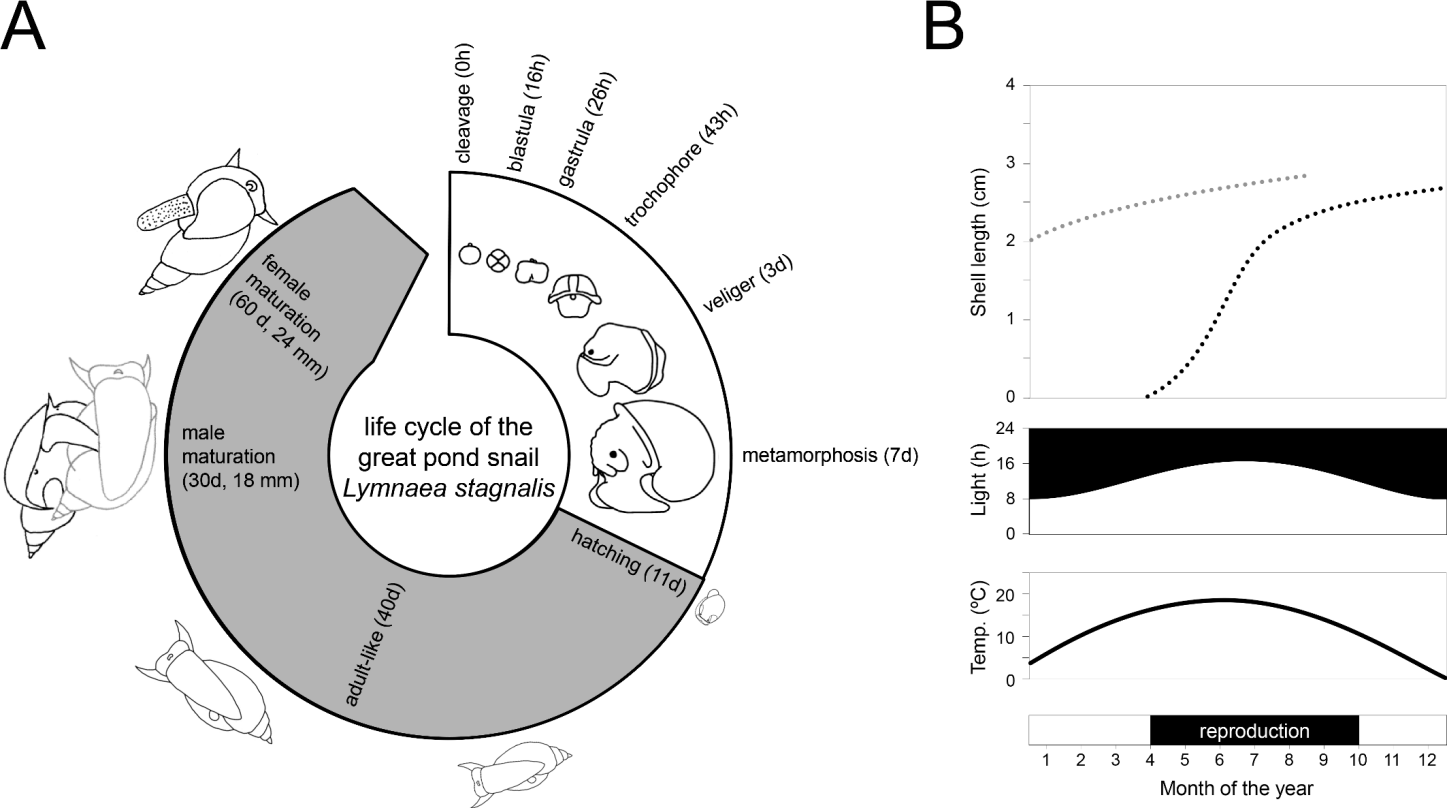
Life cycle and wild reproductive habit of *L. stagnalis*. (**A**) The embryonic development in the egg from zygote to hatching (over 11–12 days) is depicted in the white area of the life cycle and consists of six main stages: cleavage, blastula, gastrula, trochophore, veliger and metamorphosis (Source data from Ivashkin et al., 2015). The grey area of the life cycle depicts growth and development after hatching. Although *L. stagnalis* is a simultaneous hermaphrodite, the male reproductive organs are functional before the female ones (Koene and Ter Maat, 2004): specimens reach male and female maturation on average at an age of 30 and 60 days, respectively (based on Koene, 2010). (**B**) In the wild, generations only partly overlap, as depicted by the two dotted growth curves (top; based on Nakadera et al., 2015). Individuals that are born during spring and summer, overwinter as adults (light grey dotted line) after which they overlap with the adult generation of the next year (black dotted line). The external conditions such as light and temperature (middle), which strongly influence when egg laying occurs (bottom), are depicted for the situation in a typical temperate zone.

The life cycle and reproductive biology of the species are well-characterised (Ivashkin et al., 2015; Koene, 2010; Mescheryakov, 1990; Morrill, 1982; Figure 2A). Embryos develop inside transparent eggs packaged in a translucent gelatinous mass allowing observers to follow their developmental stages in detail over the 11–12 days to hatching. The time from laid eggs to mature reproductive adults can be as short as two months depending on the temperature, photoperiod, feeding regime and mate availability at the location where they are being raised. In their natural habitat, they have been found to reach an age in excess of one year, but in the laboratory they live longer, up to two years (Janse et al., 1988; Nakadera et al., 2015). For laboratory breeding, a large and genetically diverse breeding stock is recommended as this will facilitate a well-standardised stock population without too much inbreeding. The largest and longest-maintained breeding facility is found at the Vrije Universiteit in Amsterdam, where *L. stagnalis* has been bred continuously for over 50 years (Nakadera et al., 2014).

Its well-characterised embryonic and post-embryonic processes have promoted extensive use of *L. stagnalis* in the field of developmental biology. This snail has helped us to understand the mechanisms underlying shell formation (Hohagen and Jackson, 2013), the transfer of non-genetic information to the developing embryos (Ivashkin et al., 2015), and resource allocation during development (Koene and Ter Maat, 2004). Moreover, studies with *L. stagnalis* has also helped develop and evaluate models in physiology, such as the “dynamic energy budget” model (Zonneveld and Kooijman, 1989; Zimmer et al., 2014).

This snail is a simultaneous hermaphrodite, meaning that mature individuals express a functional male and female reproductive system at the same time within one body. Despite having two functional sexes, specimens copulate unilaterally; one individual plays the male role and the other the female role within one mating interaction (Hoffer et al., 2017). There is no obligate alternation of sexual roles, but when both individuals of a mating pair are motivated to mate in the male role they can perform a second copulation with the same partner in the opposite sexual role (Koene, 2010; Ter Maat et al., 2012; Van Duivenboden and Maat, 1985). An integrated laboratory and field study showed that, in the wild, most individuals are born during the spring and summer seasons and generations partly overlap because the latter cohorts overwinter and overlap with mature individuals of the new spring cohort (Figure 2B; Nakadera et al., 2015). The same study showed that both age and size significantly affected the sex role decision under laboratory conditions. This species is quite fecund in the laboratory: snails from the mass culture in Amsterdam produce a large number of offspring all year round (Nakadera et al., 2014); however, an initial field study found a more moderate fecundity rate in natural populations (Nakadera et al., 2017). In the laboratory, specimens produce on average 2–3 egg masses per week each containing 100–150 eggs, depending on the body size of the individual (Nakadera et al., 2014). The hatching rate under laboratory conditions is generally above 90% (Hoffer et al., 2017). Based on laboratory, semi-field and field studies, explicit inbreeding or self-fertilisation depression for this species have been found to be absent (Coutellec and Lagadic, 2006; Escobar et al., 2011; Koene et al., 2008; Puurtinen et al., 2007) or very unlikely (Coutellec and Caquet, 2011), however the reasons for this remain unclear (Box 1). Nevertheless, eggs are preferentially outcrossed with sperm from mating partners, which can be stored for two months, and individuals only use their own 'autosperm' when this 'allosperm' is not available (Nakadera et al., 2014).

Box 1. Outstanding questions about the natural history of the great pond snail.

- Why is inbreeding depression less strong in *L. stagnalis* than in related freshwater snail species?
- How different are long-term laboratory-bred strains from natural populations as a result of different selection pressures influencing development, mating propensity, self-fertilisation, learning and/or changes in sensitivity due to changing biotic and abiotic factors?
- How can the knowledge about host-parasite interaction be applied to control the spread of parasites in the natural habitat?
- How phenotypically plastic or evolutionarily adaptable is this species to changes in biotic and abiotic conditions in its habitat (e.g., temperature, light and/or chemical pollution, and resulting changes in ecosystem composition)?
- Why are sinistral individuals not found more often in natural populations and what does that mean for the natural selection pressures on this chiral morph?
- Are the detection and avoidance of positive and negative stimuli only present in the laboratory or is this learned behaviour also exhibited under field conditions (e.g., predicting presence of food, mating partners and/or predators)?
- How can the knowledge about the regulatory mechanisms underlying reproduction be better used to understand the evolution and flexibility of the hermaphroditic lifestyle?

## A gold standard model organism for neuroscience

The squid *Loligo forbesii* and sea hare *Aplysia californica* were the first molluscan models for examining neuronal processes. *L. stagnalis* emerged shortly afterwards, and was described as “a reductionistic, yet sophisticated model to address fundamental questions in learning and memory” (Rivi et al., 2020). Molluscs were used extensively in the field of neurobiology in the 20th century, typically because their central nervous systems were, in most cases, more accessible than those of vertebrate animals. Technical developments since then mean many such experiments can now be performed on vertebrates as well, yet we would argue that invertebrates still have substantial advantages for our understanding of the central nervous system.

**Figure 3.**
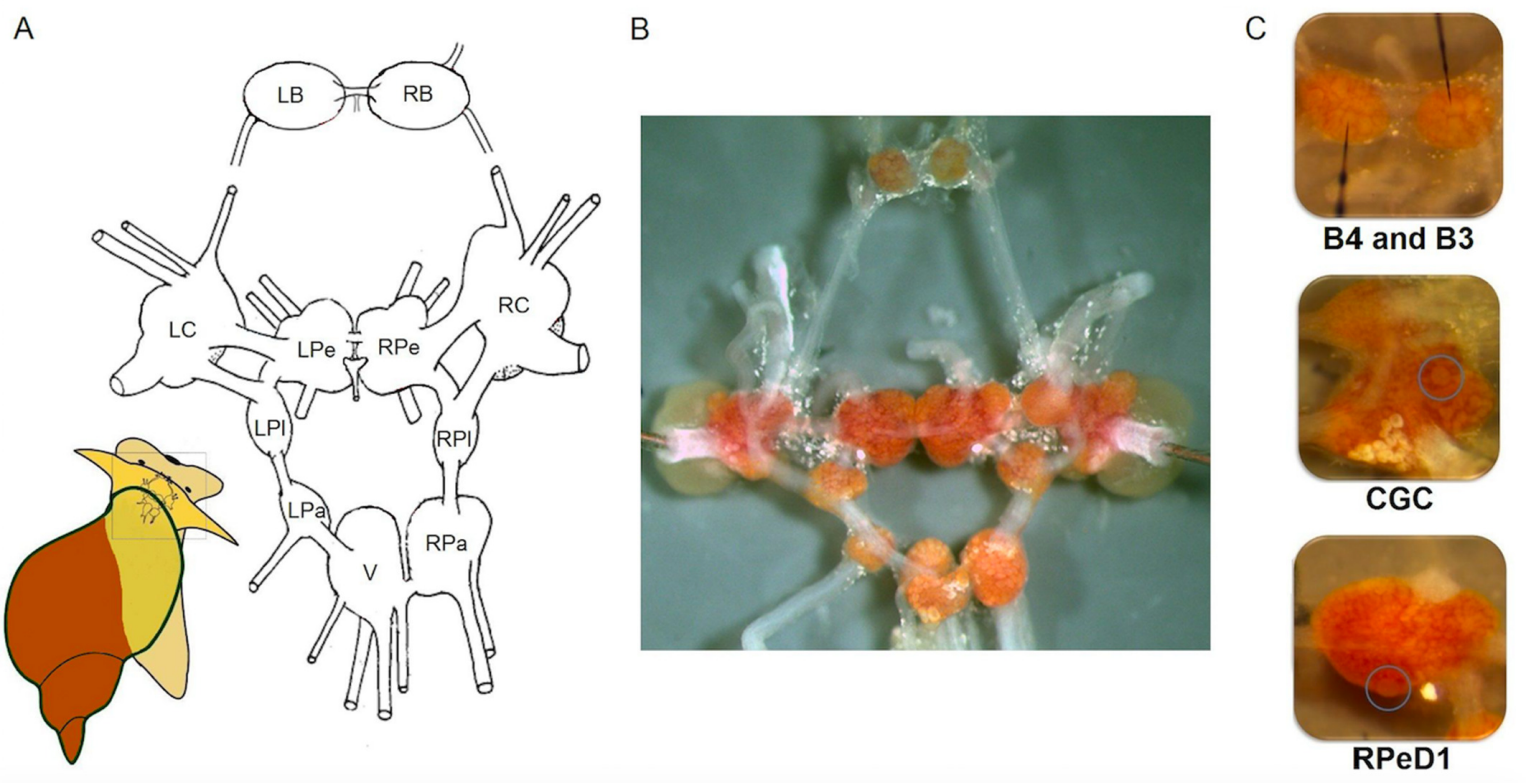
The central nervous system and identified single neurons of *L. stagnalis*. (**A**) Schematic map (dorsal view) of the isolated whole central nervous system that is formed of the paired (left and right) buccal (LB, RB), cerebral (LC, RC), pedal (LPe, RPe), pleural (LPl, RPl), parietal (LPa, RPa) and unpaired visceral (V) ganglia. (**B**) Isolated central nervous system showing the arrangement of the 11 interconnected ganglia. Brightly pigmented orange-coloured neurons are localised on the surfaces of the ganglia. (**C**) Identified single neurons: B4 (left), B3 (right; motor neurons responsible for the implementation of feeding), CGC (interneuron in cerebral ganglia modulating the feeding and learning) and RPeD1 (interneuron in pedal ganglia regulating the respiration and heartbeat).

The relatively simple central nervous system *of L. stagnalis* is organised in a ring of 11 interconnected ganglia (Figure 3A,B) with ~25,000 neurons. The neurons are mostly large (30–150 µm in diameter) and their bright, orange-coloured cell bodies are located on the surface of the ganglia (Figure 3B; Kemenes and Benjamin, 2009). Thus they are readily accessible for experimental purposes, simplifying investigations of neural clusters, circuits and even single neurons, which can be reliably identified for functional examination using a variety of approaches such as electrophysiological, molecular and analytical techniques (Crossley et al., 2018; de Hoog et al., 2019; El Filali et al., 2015; Harris et al., 2012; Kemenes et al., 2011; Lu et al., 2016; Samu et al., 2012; Wagatsuma et al., 2005; Zhang et al., 2018b).

Individual neurons (Figure 3C; Benjamin and Crossley, 2020) and their synaptic connectivity were identified as parts of circuits controlling behaviours (Audesirk et al., 1985; Benjamin, 2012; McCrohan and Benjamin, 1980a; McCrohan and Benjamin, 1980b; Syed and Winlow, 1991; Syed et al., 1990). Combining this knowledge with an understanding of the molecular mechanisms, often from laboratory studies, has helped produce an integrated picture of the processes underlying learning and memory, such as consolidation, reconsolidation, extinction and forgetting. The molecular pathways involved in memory formation in *L. stagnalis* were recently identified, providing further evidence the mechanisms of learning and memory consolidation are conserved across phylogenetic groups in a variety of learning paradigms, including non-associative or associative learning, and operant or classical conditioning (Benjamin and Kemenes, 2013; Fulton et al., 2005; Josselyn and Nguyen, 2005; Kemenes and Benjamin, 2009; Kemenes et al., 2002; Marra et al., 2013; Michel et al., 2008; Nikitin et al., 2008; Park et al., 1998; Pirger et al., 2010; Pirger et al., 2014a; Pirger et al., 2014b; Ribeiro et al., 2003; Rivi et al., 2020; Sadamoto et al., 1998; Sadamoto et al., 2010; Schacher et al., 1988; Vigil and Giese, 2018; Wan et al., 2010). Recently studies have also revealed differences in learning ability at the behavioural level between situations in the laboratory and the field (e.g., Dalesman and Lukowiak, 2012; Dalesman et al., 2015; Dalesman, 2018).

The well-characterised proximate processes at the molecular, cellular and circuit levels mean studying this simple nervous system has the potential to provide insights into how snails can respond appropriately to environmental challenges (e.g., climatic change or pharmacologically active compounds). Also, since their behaviours are generated by reflexive and central pattern generator networks similar to those of vertebrates (Katz and Hooper, 2007), results from snails offer insights into the fundamental processes important for these animals too.

Finally, recent developments have enabled this species to be used as a model for understanding the basis of neurodegenerative diseases. Comparative analyses have yielded several homologs to human genes linked to ageing and neurodegenerative diseases in *A. californica* and this species has proved well suited for studying these processes (Moroz et al., 2006; Moroz and Kohn, 2010). Similar molecular sequences have been identified in *L. stagnalis* (Fodor et al., 2020b). With the appropriate genetic background, its accessible central nervous system and relatively long and well-characterised life span mean *L. stagnalis* is highly suitable for studying the biological mechanisms of ageing, age-related memory loss and neurodegenerative diseases, such as Parkinson’s and Alzheimer’s diseases (Arundell et al., 2006; de Weerd et al., 2017; Ford et al., 2017; Hermann et al., 2007; Hermann et al., 2020; Maasz et al., 2017; Patel et al., 2006; Pirger et al., 2014b; Scutt et al., 2015; Vehovszky et al., 2007; Yeoman and Faragher, 2001; Yeoman et al., 2008).

## Ecotoxicology and risk assessment in a changing global environment

It has become clear that in the globalised world, climate change, light pollution, micro- and nanoplastics, and pharmacologically active compounds all pose a challenge to animal life. These challenges affect the availability of suitable habitats and reduce the quality of the land, lakes and rivers. They also change the environmental composition of pathogens, parasites, competitors and invaders. Understanding how global ecosystems are adapting to pollution is a complex problem; it requires researchers to monitor natural populations and conduct laboratory studies to discover the bases of adaptations or the lack thereof (Markow, 2015).

Molluscs the second most diverse animal group and considered to be excellent indicators of ecosystem health. For example, *L. stagnalis* is a sensitive and reliable species for such studies (Amorim et al., 2019), in a large part because of its well-characterised developmental and reproductive biology as described above. The major targets in this field of study have been metal-risk assessment (Crémazy et al., 2018; Pyatt et al., 1997; Vlaeminck et al., 2019), the effects of pesticides (Coutellec et al., 2008; Lance et al., 2016; Tufi et al., 2015; Vehovszky et al., 2015), nanotoxicology (Hudson et al., 2019; Stoiber et al., 2015), the development of toxicokinetic models (Baudrot et al., 2018), immunocompetence analyses (Boisseaux et al., 2018; Gust et al., 2013b), and global warming risk assessment (Leicht et al., 2017; Leicht and Seppälä, 2019; Teskey et al., 2012). Studies on *L. stagnalis* have measured toxicological values such as mortality concentrations (e.g., LC50) and impairment of reproduction (e.g., EC50) but also sub-lethal and more sensitive endpoints such as reproductive success, growth, cellular and molecular biomarkers that may be coupled with behavioural responses (Amorim et al., 2019).

*L. stagnalis* has also been recognised as a useful organism to examine the effects of pharmacologically active compounds and micro- and nanoplastics on aquatic organisms (Amorim et al., 2019; Charles et al., 2016; Ducrot et al., 2014; Gust et al., 2013a; Horton et al., 2020; Pirger et al., 2018; Zrinyi et al., 2017). However, it must be pointed out that researchers need to be critical of the generalisability of results while performing such experiments since there are differences between the endocrine system of molluscs and vertebrates; molluscs, for example, do not have functional oestrogen receptors (Eick and Thornton, 2011; Lagadic et al., 2007; Scott, 2012). It is also important to recognise that molluscs are not suitable for some types of ecotoxicological studies and they cannot always substitute for fish.

Notably, *L. stagnalis* is the first aquatic non-arthropod invertebrate model organism to be recognised in environmental risk assessments. The developed standard reproduction test was officially approved by the national coordinators of the Organisation for Economic Cooperation and Development (OECD, 2016) thus paving the way for investigating ecotoxicological effects in more detail. Such information will contribute to a more complete picture of the mode of action of potentially toxic substances and other environmental factors and provide assessments of risk for individual species of different types and wider ecosystems.

## Combining evolution and natural history

Within the field of evolutionary biology, *L. stagnalis* has helped us to understand the evolution of several phenomena. Left-right asymmetry is a general evolutionary phenomenon seen across a variety of species, including humans where the congenital condition situs inversus results in the mirrored position and shape of the heart and liver (Blum et al., 2014; Oliverio et al., 2010; Palmer, 2009; Palmer, 2016). The coil or chirality of snail shells is one of the more spectacular outward manifestations of this asymmetry. Snails found in nature can have shells that coil either to the right or left, with most species being right-coiling. Specimens of *L. stagnalis* that coil in the opposite left-winding, or sinistral, direction are rare and often categorised as 'unlucky' because their different chirality makes it difficult for them to mate the more usual, right-winding individuals (Davison et al., 2009). Left-winding snails are also less able to learn in a mate-choice context (Koene and Cosijn, 2012). The existence of the two different morphs has made this species ideal for studying chiromorphogenesis, i.e. the first step of left-right symmetry breaking. Genes and signalling pathways that are responsible for snail coiling have been identified (Abe et al., 2014; Davison et al., 2016; Kuroda, 2014; Kuroda, 2015; Kuroda et al., 2016), and similar signalling pathways are required for vertebrate chiromorphogenesis as well (Kuroda, 2015). Studies on *L. stagnalis* can give important insights into the evolution of body plans in other phyla, and may have wider medical implications, including an understanding of situs inversus.

*L. stagnalis* has also played a crucial role in studies into the evolution of hermaphroditism and its consequences for sexual selection. This area of research relies heavily on a solid understanding of the natural history of this species. Selection of sexual traits that affect mating success was previously considered not to act in simultaneous hermaphrodites (Charnov, 1979; Darwin, 1871; Greeff and Michiels, 1999; Morgan, 1994). However, recent research, including work with *L. stagnalis*, has contradicted this earlier conclusion (Anthes et al., 2010; Baur, 1998; Chase, 2007; Janicke et al., 2016; Michiels, 1998; Nakadera and Koene, 2013). Unilateral mating of *L. stagnalis* offered a unique opportunity to test whether sexual selection acts independently on the two sexual roles of a simultaneous hermaphrodite (Anthes et al., 2010; Arnold, 1994; Hoffer et al., 2017). Recent experiments have also revealed that male and female reproductive strategies can be optimised independently in this species. This was done by measuring sexual selection gradients (also called Bateman gradients), which reveal the relationship between the number of matings and the reproductive success of the sexual functions (Anthes et al., 2010). Experiments with *L. stagnalis* showed that this mating system seems largely male-driven, and that the sexual selection gradients are consistently positive for the male function but change over time to benefit the opposite sex (Anthes et al., 2010; Hoffer et al., 2017; Janicke et al., 2016; Pélissié et al., 2012). These pioneering works, which measured and quantified the processes of sexual selection and their underlying mechanisms, thus incorporated this hermaphrodite into the general Darwin-Bateman paradigm that had so far mainly been tested on separate-sexed species. They also described both the evolutionary potential and limitations of hermaphrodite animals and revealed important practical applications for the conservation of wildlife.

## New opportunities from a growing multi-omics coverage

From about 1980, continued attention was given to the physiological characterisation of *L. stagnalis*, but more recent research has focussed on an 'omics' approach to better understand the underlying molecular processes (Santama et al., 1993; Santama et al., 1995a; Santama et al., 1995b; Santama and Benjamin, 2000). Due to its pre-eminence as a model system in neuroscience, early molecular studies tended to focus on the central nervous system (Feng et al., 2009; Johnson and Davison, 2019). The favourable anatomical features enabled the accumulation of peptidomic data from the mass spectrometry of single neurons (Perry et al., 1999; Worster et al., 1998), making the neuropeptidergic system the most intensely studied part of the central nervous system (Buckett et al., 1990; Perry et al., 1998). Taking advantage of a variety of platforms available for nucleotide sequencing: Sanger (Davison and Blaxter, 2005; Sadamoto et al., 2004; Swart et al., 2019), Illumina (Korneev et al., 2018; Sadamoto et al., 2012; Stewart et al., 2016), BGISEQ (Jehn et al., 2018) and Oxford Nanopore (Fodor et al., 2020a), many sequencing methodologies have been successfully applied to this species.

Extensive genomic, transcriptomic and peptidomic data for *L. stagnalis* are available in the NCBI database. Four major transcriptome datasets were established by sequencing mRNA from the central nervous system (Bouétard et al., 2012; Davison and Blaxter, 2005; Feng et al., 2009; Sadamoto et al., 2012), and then used to identify genes and proteins, thus providing a solid genetic background for *L. stagnalis*. Furthermore, an unannotated draft genome is already available and a collaborative effort is underway to produce an annotated genome (Johnson and Davison, 2019) which would largely solve the problem of the lack of molecular information that has so far inhibited research in the *L. stagnalis* model system (Rivi et al., 2020). Approximately 100 (neuro)peptides have been identified so far (Benjamin and Kemenes, 2020), encoded by genes involved in various regulatory processes (Table 1). These findings contributed to a global understanding of the natural history of *L. stagnalis* by characterising the molecular and cellular processes underlying chirality, reproduction, immune processes, host-parasite interaction, and acute and chronic adaptive responses to toxic substances in the environment.

**Table 1.** List of some of the most important (neuro)peptides identified in *L. stagnalis.*

Molecule	Abbreviation	Function	Accession number	Reference
caudodorsal cell hormones	CDCH	reproduction	P06308	Vreugdenhil et al., 1988
FMRFamides	FMRF	reproduction, cardiac control	P19802	Linacre et al., 1990
conopressin	-	reproduction	AAB35220	Van Kesteren et al., 1995
neuropeptide Y	NPY	reproduction, development	CAB63265	De Jong-Brink et al., 1999
actin-related diaphanous genes (1, 2)	dia 1, dia 2	development, chirality	KX387869, KX387870 KX387871, KX387872	Kuroda et al., 2016
insulin-related peptides (I, II, III, V, VII)	MIPs	development	CAA41989; P25289; AAB28954; AAA09966; AAB46831	Smit et al., 1991; Smit et al., 1992; Smit et al., 1993b; Smit et al., 1996; Smit et al., 1998
sodium stimulating hormone	SIS	ion and water control	P42579	Smit et al., 1993a
small cardioactive peptide	SCP	feeding, cardiac control	AAC99318	Perry et al., 1999
myomodulin	MIP	feeding, cardiac control	CAA65635	Kellett et al., 1996
pituitary adenylate cyclase-activating polypeptide-like molecule	PACAP-like	learning and memory	-	Pirger et al., 2010
cAMP response element-binding proteins (1, 2)	CREB 1 CREB 2	learning and memory	AB041522; AB083656	Sadamoto et al., 2004
glutathione reductase and peroxidase	Gred Gpx	metabolic detoxification	FJ418794, FJ418796	Bouétard et al., 2014
catalase	CAT	metabolic detoxification	FJ418795	Bouétard et al., 2014
superoxide dismutase	SOD	metabolic detoxification	AY332385	Zelck et al., 2005
heat-shock protein	HSP70	stress response	DQ206432	Fei et al., 2007
molluscan defence molecule	MDM	immune system	AAC47132	Hoek et al., 1996
allograft inflammatory factor-1	AIF-1	immune system	DQ278446	van Kesteren et al., 2006

Furthermore, the CRISPR/Cas9 genome editing method has recently been applied to molluscs (Henry and Lyons, 2016; Perry and Henry, 2015). In *L. stagnalis,* it was used to knock out the gene responsible for coiling direction during development, leading to a better understanding of chirality in the life of the two morphs (Abe and Kuroda, 2019). The establishment of genome editing in *L. stagnalis* opens up significant opportunities for functional genomics to investigate the role of specific genes, for example, in snail developmental, toxicology and immunobiological studies.

## Conclusion

Research on model organisms has been essential to developing the current understanding of how life works. The unique features of *L. stagnalis* make it an excellent experimental system to complement the classic invertebrate (*C. elegans, D. melanogaster*) and vertebrate (*D. rerio, M. musculu*s) models. Research utilising this species is expected to lead to future breakthroughs in a number of scientific fields, especially in neuroscience and evolutionary biology. For example, as a simultaneously hermaphroditic outcrossing species, it presents the opportunity to test the generality of hypotheses that are mainly based on non-hermaphroditic or self-fertilising models. There is considerable information about the natural history of *L. stagnalis* compared to some other model species, but we feel some areas of research using *L. stagnalis* – in particular neurobiology and ecotoxicology – would benefit by extending more of their studies out of the laboratory and into the field. We believe that a deeper integration of information from field studies with input from laboratory findings – such as applying experimental designs and approaches developed in the laboratory to populations in the wild – will provide future opportunities for further innovation (Box 2). Such efforts could address the unanswered questions regarding this model organism (see Box 1). Significantly, emerging recent technical approaches such as pocket-sized sequencing devices, especially with their impending breakthrough also in protein sequencing, start allowing researchers to perform more experiments in the field such as following molecular mechanisms of learning.

Box 2. How can findings at different biological levels be integrated to better understand this species’ natural history?

- It needs to be established at what level *L. stagnalis* can function as a model for medical research such as neurodegenerative disease and be a substitute for standard vertebrate models. This requires a better understanding of how such functions affect this species in its natural habitat.
- The new molecular techniques and available 'omics' data provide an incentive for research that aims to understand the mechanisms underlying natural history processes such as sex allocation, simultaneous hermaphroditism, reproductive success, chirality and learning.
- The knowledge about learning and decision-making in the laboratory needs to be extended to field populations to promote future developments in, for example, neural network-inspired robotics.

## Data Availability

All data generated during the preparation of this review are included in the manuscript.

## References

[bib1] Abe M, Takahashi H, Kuroda R (2014). Spiral cleavages determine the left-right body plan by regulating nodal pathway in Monomorphic Gastropods, *Physa acuta*. The International Journal of Developmental Biology.

[bib2] Abe M, Kuroda R (2019). The development of CRISPR for a mollusc establishes the formin *Lsdia1* as the long-sought gene for snail dextral/sinistral coiling. Development.

[bib3] Adema CM, van Deutekom-Mulder EC, van der Knaap WP, Sminia T (1994). Schistosomicidal activities of *Lymnaea stagnalis* haemocytes: the role of oxygen radicals. Parasitology.

[bib4] Amorim J, Abreu I, Rodrigues P, Peixoto D, Pinheiro C, Saraiva A, Carvalho AP, Guimarães L, Oliva-Teles L (2019). *Lymnaea stagnalis* as a freshwater model invertebrate for ecotoxicological studies. Science of the Total Environment.

[bib5] Anthes N, David P, Auld JR, Hoffer JN, Jarne P, Koene JM, Kokko H, Lorenzi MC, Pélissié B, Sprenger D, Staikou A, Schärer L (2010). Bateman gradients in hermaphrodites: an extended approach to quantify sexual selection. The American Naturalist.

[bib6] Arnold SJ (1994). Bateman's Principles and the Measurement of Sexual Selection in Plants and Animals. The American Naturalist.

[bib7] Arundell M, Patel BA, Straub V, Allen MC, Janse C, O'Hare D, Parker K, Gard PR, Yeoman MS (2006). Effects of age on feeding behavior and chemosensory processing in the pond snail, *Lymnaea stagnalis*. Neurobiology of Aging.

[bib8] Atli G, Grosell M (2016). Characterization and response of antioxidant systems in the tissues of the freshwater pond snail (*Lymnaea stagnalis*) during acute copper exposure. Aquatic Toxicology.

[bib9] Audesirk G, Audesirk T, McCaman RE, Ono JK (1985). Evidence for genetic influences on neurotransmitter content of identified neurones of *Lymnaea stagnalis*. Comparative Biochemistry and Physiology Part C: Comparative Pharmacology.

[bib10] Baudrot V, Preux S, Ducrot V, Pave A, Charles S (2018). New insights to compare and choose TKTD models for survival based on an interlaboratory study for *Lymnaea stagnalis* exposed to cd. Environmental Science & Technology.

[bib11] Baur B, TRBaAP M (1998). Sperm competition in molluscs. Sperm Competition and Sexual Selection.

[bib12] Benjamin P (2008). Lymnaea. Scholarpedia.

[bib13] Benjamin PR (2012). Distributed network organization underlying feeding behavior in the mollusk Lymnaea. Neural Systems & Circuits.

[bib14] Benjamin PR, Crossley M (2020). Gastropod Feeding Systems: Evolution of Neural Circuits That Generate Diverse Behaviors.

[bib15] Benjamin P, Kemenes I (2013). *Lymnaea* neuropeptide genes. Scholarpedia.

[bib16] Benjamin PR, Kemenes I, Saleuddin A. B. L, Orchard I (2020). Peptidergic systems in the pond snail *Lymnaea*: From genes to hormones and behavior. Advances in Invertebrate (Neuro) Endocrinology.

[bib17] Blum M, Feistel K, Thumberger T, Schweickert A (2014). The evolution and conservation of left-right patterning mechanisms. Development.

[bib18] Boisseaux P, Noury P, Delorme N, Perrier L, Thomas-Guyon H, Garric J (2018). Immunocompetence analysis of the aquatic snail *Lymnaea stagnalis* exposed to urban wastewaters. Environmental Science and Pollution Research.

[bib19] Bouétard A, Noirot C, Besnard AL, Bouchez O, Choisne D, Robe E, Klopp C, Lagadic L, Coutellec MA (2012). Pyrosequencing-based transcriptomic resources in the pond snail *Lymnaea stagnalis*, with a focus on genes involved in molecular response to diquat-induced stress. Ecotoxicology.

[bib20] Bouétard A, Côte J, Besnard AL, Collinet M, Coutellec MA (2014). Environmental versus anthropogenic effects on population adaptive divergence in the freshwater snail *Lymnaea stagnalis*. PLOS ONE.

[bib21] Buckett KJ, Peters M, Dockray GJ, Van Minnen J, Benjamin PR (1990). Regulation of heartbeat in *Lymnaea* by motoneurons containing FMRFamide-like peptides. Journal of Neurophysiology.

[bib22] Charles S, Ducrot V, Azam D, Benstead R, Brettschneider D, De Schamphelaere K, Filipe Goncalves S, Green JW, Holbech H, Hutchinson TH, Faber D, Laranjeiro F, Matthiessen P, Norrgren L, Oehlmann J, Reategui-Zirena E, Seeland-Fremer A, Teigeler M, Thome JP, Tobor Kaplon M, Weltje L, Lagadic L (2016). Optimizing the design of a reproduction toxicity test with the pond snail *Lymnaea stagnalis*. Regulatory Toxicology and Pharmacology.

[bib23] Charnov EL (1979). Simultaneous hermaphroditism and sexual selection. PNAS.

[bib24] Chase R (2007). The function of dart shooting in helicid snails*. American Malacological Bulletin.

[bib25] Coutellec MA, Delous G, Cravedi JP, Lagadic L (2008). Effects of the mixture of diquat and a nonylphenol polyethoxylate adjuvant on fecundity and progeny early performances of the pond snail *Lymnaea stagnalis* in laboratory bioassays and microcosms. Chemosphere.

[bib26] Coutellec MA, Caquet T (2011). Heterosis and inbreeding depression in bottlenecked populations: a test in the hermaphroditic freshwater snail *Lymnaea stagnalis*. Journal of Evolutionary Biology.

[bib27] Coutellec MA, Lagadic L (2006). Effects of self-fertilization, environmental stress and exposure to xenobiotics on fitness-related traits of the freshwater snail *Lymnaea stagnalis*. Ecotoxicology.

[bib28] Crémazy A, Brix KV, Wood CM (2018). Chronic toxicity of binary mixtures of six metals (Ag, cd, cu, ni, pb, and zn) to the great pond snail *Lymnaea stagnalis*. Environmental Science & Technology.

[bib29] Crossley M, Staras K, Kemenes G (2018). A central control circuit for encoding perceived food value. Science Advances.

[bib30] Dalesman S, Rendle A, Dall SR (2015). Habitat stability, predation risk and 'memory syndromes'. Scientific Reports.

[bib31] Dalesman S (2018). Habitat and social context affect memory phenotype, exploration and covariance among these traits. Philosophical Transactions of the Royal Society B: Biological Sciences.

[bib32] Dalesman S, Lukowiak K (2012). How stress alters memory in 'smart' snails. PLOS ONE.

[bib33] Darwin C (1871). The Descent of Man and Selection in Relation to Sex.

[bib34] Davison A, Barton NH, Clarke B (2009). The effect of coil phenotypes and genotypes on the fecundity and viability of Partula suturalis and *Lymnaea stagnalis*: implications for the evolution of sinistral snails. Journal of Evolutionary Biology.

[bib35] Davison A, McDowell GS, Holden JM, Johnson HF, Koutsovoulos GD, Liu MM, Hulpiau P, Van Roy F, Wade CM, Banerjee R, Yang F, Chiba S, Davey JW, Jackson DJ, Levin M, Blaxter ML (2016). Formin is associated with Left-Right asymmetry in the pond snail and the frog. Current Biology.

[bib36] Davison A, Blaxter ML (2005). An expressed sequence tag survey of gene expression in the pond snail *Lymnaea stagnalis*, an intermediate vector of Trematodes [corrected]. Parasitology.

[bib37] de Hoog E, Lukewich MK, Spencer GE (2019). Retinoid receptor-based signaling plays a role in voltage-dependent inhibition of invertebrate voltage-gated Ca^2+^ channels. Journal of Biological Chemistry.

[bib38] De Jong-Brink M, Reid CN, Tensen CP, Ter Maat A (1999). Parasites flicking the NPY gene on the host's switchboard: why NPY?. The FASEB Journal.

[bib39] de Weerd L, Hermann PM, Wildering WC (2017). Linking the 'why' and 'how' of ageing: evidence for somatotropic control of long-term memory function in the pond snail *Lymnaea stagnalis*. The Journal of Experimental Biology.

[bib40] Ducrot V, Askem C, Azam D, Brettschneider D, Brown R, Charles S, Coke M, Collinet M, Delignette-Muller ML, Forfait-Dubuc C, Holbech H, Hutchinson T, Jach A, Kinnberg KL, Lacoste C, Le Page G, Matthiessen P, Oehlmann J, Rice L, Roberts E, Ruppert K, Davis JE, Veauvy C, Weltje L, Wortham R, Lagadic L (2014). Development and validation of an OECD reproductive toxicity test guideline with the pond snail *Lymnaea stagnalis* (Mollusca, gastropoda). Regulatory Toxicology and Pharmacology.

[bib41] Eick GN, Thornton JW (2011). Evolution of steroid receptors from an estrogen-sensitive ancestral receptor. Molecular and Cellular Endocrinology.

[bib42] El Filali Z, de Boer PA, Pieneman AW, de Lange RP, Jansen RF, Ter Maat A, van der Schors RC, Li KW, van Straalen NM, Koene JM (2015). Single-cell analysis of peptide expression and electrophysiology of right parietal neurons involved in male copulation behavior of a simultaneous hermaphrodite. Invertebrate Neuroscience.

[bib43] Ericsson AC, Crim MJ, Franklin CL (2013). A brief history of animal modeling. Missouri Medicine.

[bib44] Escobar JS, Auld JR, Correa AC, Alonso JM, Bony YK, Coutellec M-A, Koene JM, Pointier J-P, Jarne P, David P (2011). Patterns of mating-system evolution in hermaphroditic animals: correlations among selfing rate, inbreeding depression, and the timing of reproduction. Evolution.

[bib45] Fei G, Guo C, Sun HS, Feng ZP (2007). Chronic hypoxia stress-induced differential modulation of heat-shock protein 70 and presynaptic proteins. Journal of Neurochemistry.

[bib46] Feng ZP, Zhang Z, van Kesteren RE, Straub VA, van Nierop P, Jin K, Nejatbakhsh N, Goldberg JI, Spencer GE, Yeoman MS, Wildering W, Coorssen JR, Croll RP, Buck LT, Syed NI, Smit AB (2009). Transcriptome analysis of the central nervous system of the mollusc *Lymnaea stagnalis*. BMC Genomics.

[bib47] Ferté H, Depaquit J, Carré S, Villena I, Léger N (2005). Presence of *Trichobilharzia szidati* in *Lymnaea stagnalis* and *T. franki* in *Radix auricularia* in northeastern france: molecular evidence. Parasitology Research.

[bib48] Fodor I, Zrinyi Z, Urban P, Herczeg R, Buki G, Koene JM, Tsai PS, Pirger Z (2020a). Identification presence and possible multifunctional regulatory role of invertebrate gonadotropin-releasing hormone/corazonin molecule in the great pond snail (*Lymnaea stagnalis*). bioRxiv.

[bib49] Fodor I, Urban P, Kemenes G, Koene JM, Pirger Z (2020b). Aging and Disease-Relevant Gene Products in the Neuronal Transcriptome of the Great Pond Snail (Lymnaea Stagnalis): A Potential Model of Aging, Age-Related Memory Loss, and Neurodegenerative Diseases.

[bib50] Ford L, Crossley M, Vadukul DM, Kemenes G, Serpell LC (2017). Structure-dependent effects of amyloid-β on long-term memory in *Lymnaea stagnalis*. FEBS Letters.

[bib51] Frézal L, Félix M-A (2015). *C. elegans* outside the petri dish. eLife.

[bib52] Fulton D, Kemenes I, Andrew RJ, Benjamin PR (2005). A single time-window for protein synthesis-dependent long-term memory formation after one-trial appetitive conditioning. European Journal of Neuroscience.

[bib53] GBIF Secretariat (2019). GBIF Backbone Taxonomy.

[bib54] Greeff JM, Michiels NK (1999). Sperm digestion and reciprocal sperm transfer can drive hermaphrodite sex allocation to equality. The American Naturalist.

[bib55] Gust M, Fortier M, Garric J, Fournier M, Gagné F (2013a). Effects of short-term exposure to environmentally relevant concentrations of different pharmaceutical mixtures on the immune response of the pond snail *Lymnaea stagnalis*. Science of the Total Environment.

[bib56] Gust M, Fortier M, Garric J, Fournier M, Gagné F (2013b). Immunotoxicity of surface waters contaminated by municipal effluents to the snail *Lymnaea stagnalis*. Aquatic Toxicology.

[bib57] Harris CA, Buckley CL, Nowotny T, Passaro PA, Seth AK, Kemenes G, O'Shea M (2012). Multi-neuronal refractory period adapts centrally generated behaviour to reward. PLOS ONE.

[bib58] Henry JQ, Lyons DC (2016). Molluscan models: *crepidula fornicata*. Current Opinion in Genetics & Development.

[bib59] Hermann PM, Lee A, Hulliger S, Minvielle M, Ma B, Wildering WC (2007). Impairment of long-term associative memory in aging snails (*Lymnaea stagnalis*). Behavioral Neuroscience.

[bib60] Hermann PM, Perry AC, Hamad I, Wildering WC (2020). Physiological and pharmacological characterization of a molluscan neuronal efflux transporter; evidence for age-related transporter impairment. The Journal of Experimental Biology.

[bib61] Hilgers L, Schwarzer J (2019). The untapped potential of medaka and its wild relatives. eLife.

[bib62] Hodgkin AL, Huxley AF (1952). A quantitative description of membrane current and its application to conduction and excitation in nerve. The Journal of Physiology.

[bib63] Hoek RM, Smit AB, Frings H, Vink JM, de Jong-Brink M, Geraerts WP (1996). A new Ig-superfamily member, molluscan defence molecule (MDM) from *Lymnaea stagnalis*, is down-regulated during parasitosis. European Journal of Immunology.

[bib64] Hoffer JN, Mariën J, Ellers J, Koene JM (2017). Sexual selection gradients change over time in a simultaneous hermaphrodite. eLife.

[bib65] Hohagen J, Jackson DJ (2013). An ancient process in a modern mollusc: early development of the shell in *Lymnaea stagnalis*. BMC Developmental Biology.

[bib66] Horton AA, Newbold LK, Palacio-Cortés AM, Spurgeon DJ, Pereira MG, Carter H, Gweon HS, Vijver MG, van Bodegom PM, Navarro da Silva MA, Lahive E (2020). Accumulation of polybrominated diphenyl ethers and microbiome response in the great pond snail *Lymnaea stagnalis* with exposure to nylon (polyamide) microplastics. Ecotoxicology and Environmental Safety.

[bib67] Hudson ML, Costello DM, Daley JM, Burton GA (2019). Species-Specific (*Hyalella azteca* and *lymnea stagnalis*) Dietary accumulation of gold Nano-particles associated with periphyton. Bulletin of Environmental Contamination and Toxicology.

[bib68] Ivashkin E, Khabarova MY, Melnikova V, Nezlin LP, Kharchenko O, Voronezhskaya EE, Adameyko I (2015). Serotonin mediates maternal effects and directs developmental and behavioral changes in the progeny of snails. Cell Reports.

[bib69] Janicke T, Häderer IK, Lajeunesse MJ, Anthes N (2016). Darwinian sex roles confirmed across the animal kingdom. Science Advances.

[bib70] Janse C, Slob W, Popelier CM, Vogelaar JW (1988). Survival characteristics of the mollusc *Lymnaea stagnalis* under constant culture conditions: effects of aging and disease. Mechanisms of Ageing and Development.

[bib71] Jehn J, Gebert D, Pipilescu F, Stern S, Kiefer JST, Hewel C, Rosenkranz D (2018). *PIWI* genes and piRNAs are ubiquitously expressed in mollusks and show patterns of lineage-specific adaptation. Communications Biology.

[bib72] Johnson HF, Davison A (2019). A new set of endogenous control genes for use in quantitative real-time PCR experiments show that formin Ldia2dex transcripts are enriched in the early embryo of the pond snail *Lymnaea stagnalis* (Panpulmonata). Journal of Molluscan Studies.

[bib73] Josselyn SA, Nguyen PV (2005). CREB, synapses and memory disorders: past progress and future challenges. Current Drug Targets. CNS and Neurological Disorders.

[bib74] Katz PS, Hooper SL, Greenspan G. N. R. J (2007). Invertebrate central pattern generators. Invertebrate Neurobiology.

[bib75] Kellett E, Perry SJ, Santama N, Worster BM, Benjamin PR, Burke JF (1996). Myomodulin gene of *Lymnaea*: structure, expression, and analysis of neuropeptides. The Journal of Neuroscience.

[bib76] Kemenes I, Kemenes G, Andrew RJ, Benjamin PR, O'Shea M (2002). Critical time-window for NO-cGMP-dependent long-term memory formation after one-trial appetitive conditioning. The Journal of Neuroscience.

[bib77] Kemenes I, Marra V, Crossley M, Samu D, Staras K, Kemenes G, Nowotny T (2011). Dynamic clamp with StdpC software. Nature Protocols.

[bib78] Kemenes G, Benjamin PR (2009). Lymnaea. Current Biology.

[bib79] Koene JM, Loose MJ, Wolters L (2008). Mate choice is not affected by mating history in the simultaneously hermaphroditic snail *Lymnaea stagnalis*. Journal of Molluscan Studies.

[bib80] Koene JM (2010). Neuro-endocrine control of reproduction in hermaphroditic freshwater snails: mechanisms and evolution. Frontiers in Behavioral Neuroscience.

[bib81] Koene JM, Cosijn J (2012). Twisted sex in an hermaphrodite: mirror-image mating behaviour is not learned. Journal of Molluscan Studies.

[bib82] Koene JM, Ter Maat A (2004). Energy budgets in the simultaneously hermaphroditic pond snail, *Lymnaea stagnalis*: a trade-off between growth and reproduction during development. Belgian Journal of Zoology.

[bib83] Korneev SA, Vavoulis DV, Naskar S, Dyakonova VE, Kemenes I, Kemenes G (2018). A CREB2-targeting microRNA is required for long-term memory after single-trial learning. Scientific Reports.

[bib84] Kryukova NA, Yurlova NI, Rastyagenko NM, Antonova EV, Glupov VV (2014). The influence of Plagiorchis mutationis larval infection on the cellular immune response of the snail host *Lymnaea stagnalis*. Journal of Parasitology.

[bib85] Kupfermann I, Kandel ER (1969). Neuronal controls of a behavioral response mediated by the abdominal ganglion of *Aplysia*. Science.

[bib86] Kuroda R (2014). How a single gene twists a snail. Integrative and Comparative Biology.

[bib87] Kuroda R (2015). A twisting story: how a single gene twists a snail? mechanogenetics. Quarterly Reviews of Biophysics.

[bib88] Kuroda R, Fujikura K, Abe M, Hosoiri Y, Asakawa S, Shimizu M, Umeda S, Ichikawa F, Takahashi H (2016). Diaphanous gene mutation affects spiral cleavage and chirality in snails. Scientific Reports.

[bib89] Lagadic L, Coutellec MA, Caquet T (2007). Endocrine disruption in aquatic pulmonate molluscs: few evidences, many challenges. Ecotoxicology.

[bib90] Lance E, Brient L, Bormans M, Gérard C (2006). Interactions between cyanobacteria and gastropods I. ingestion of toxic *Planktothrix agardhii* by *Lymnaea stagnalis* and the kinetics of microcystin bioaccumulation and detoxification. Aquatic Toxicology.

[bib91] Lance E, Desprat J, Holbech BF, Gérard C, Bormans M, Lawton LA, Edwards C, Wiegand C (2016). Accumulation and detoxication responses of the gastropod *Lymnaea stagnalis* to single and combined exposures to natural (cyanobacteria) and anthropogenic (the herbicide RoundUpFlash) stressors. Aquatic Toxicology.

[bib92] Langeloh L, Behrmann-Godel J, Seppälä O (2017). Natural selection on immune defense: a field experiment. Evolution.

[bib93] Langeloh L, Seppälä O (2018). Relative importance of chemical attractiveness to parasites for susceptibility to trematode infection. Ecology and Evolution.

[bib94] Leicht K, Seppälä K, Seppälä O (2017). Potential for adaptation to climate change: family-level variation in fitness-related traits and their responses to heat waves in a snail population. BMC Evolutionary Biology.

[bib95] Leicht K, Seppälä O (2019). Direct and transgenerational effects of an experimental heatwave on early life stages in a freshwater snail. Freshwater Biology.

[bib96] Linacre A, Kellett E, Saunders S, Bright K, Benjamin PR, Burke JF (1990). Cardioactive neuropeptide Phe-Met-Arg-Phe-NH_2_ (FMRFamide) and novel related peptides are encoded in multiple copies by a single gene in the snail *Lymnaea stagnalis*. The Journal of Neuroscience.

[bib97] Lu TZ, Kostelecki W, Sun CL, Dong N, Pérez Velázquez JL, Feng ZP (2016). High sensitivity of spontaneous spike frequency to sodium leak current in a *Lymnaea* pacemaker neuron. The European Journal of Neuroscience.

[bib98] Lukowiak K, Ringseis E, Spencer G, Wildering W, Syed N (1996). Operant conditioning of aerial respiratory behaviour in *Lymnaea stagnalis*. The Journal of Experimental Biology.

[bib99] Maasz G, Zrinyi Z, Reglodi D, Petrovics D, Rivnyak A, Kiss T, Jungling A, Tamas A, Pirger Z (2017). Pituitary adenylate cyclase-activating polypeptide (PACAP) has a neuroprotective function in dopamine-based neurodegeneration in rat and snail parkinsonian models. Disease Models & Mechanisms.

[bib100] Markow TA (2015). The secret lives of *Drosophila* flies. eLife.

[bib101] Marra V, O'Shea M, Benjamin PR, Kemenes I (2013). Susceptibility of memory consolidation during lapses in recall. Nature Communications.

[bib102] McCrohan CR, Benjamin PR (1980a). Synaptic relationships of the cerebral giant cells with motoneurones in the feeding system of *Lymnaea stagnalis*. The Journal of Experimental Biology.

[bib103] McCrohan CR, Benjamin PR (1980b). Patterns of activity and axonal projections of the cerebral giant cells of the snail, *Lymnaea stagnalis*. The Journal of Experimental Biology.

[bib104] Mescheryakov VN, Detlaf D. A, Vassetzky S. G (1990). The common pond snail *Lymnaea stagnalis*. Animal Species for Developmental Studies,.

[bib105] Michel M, Kemenes I, Müller U, Kemenes G (2008). Different phases of long-term memory require distinct temporal patterns of PKA activity after single-trial classical conditioning. Learning & Memory.

[bib106] Michiels NK, Trbap M (1998). Mating conflicts and sperm competition in simultaneous hermaphrodites. Sperm Competition and Sexual Selection.

[bib107] Morgan MT (1994). Models of sexual selection in hermaphrodites, especially plants. The American Naturalist.

[bib108] Moroz LL, Edwards JR, Puthanveettil SV, Kohn AB, Ha T, Heyland A, Knudsen B, Sahni A, Yu F, Liu L, Jezzini S, Lovell P, Iannucculli W, Chen M, Nguyen T, Sheng H, Shaw R, Kalachikov S, Panchin YV, Farmerie W, Russo JJ, Ju J, Kandel ER (2006). Neuronal transcriptome of *Aplysia*: neuronal compartments and circuitry. Cell.

[bib109] Moroz LL, Kohn AB (2010). Do different neurons age differently? direct genome-wide analysis of aging in single identified cholinergic neurons. Frontiers in Aging Neuroscience.

[bib110] Morrill JB, Harrison F. W, Cowden R. R (1982). Developmental biology of the freshwater invertebrates. Development of the Pulmonate Gastropod, Lymnaea.

[bib111] Nakadera Y, Blom C, Koene JM (2014). Duration of sperm storage in the simultaneous hermaphrodite *Lymnaea stagnalis*. Journal of Molluscan Studies.

[bib112] Nakadera Y, Swart EM, Maas JP, Montagne-Wajer K, Ter Maat A, Koene JM (2015). Effects of age, size, and mating history on sex role decision of a simultaneous hermaphrodite. Behavioral Ecology.

[bib113] Nakadera Y, Mariën J, Van Straalen NM, Koene JM (2017). Multiple mating in natural populations of a simultaneous hermaphrodite, *Lymnaea stagnalis*. Journal of Molluscan Studies.

[bib114] Nakadera Y, Koene JM (2013). Reproductive strategies in hermaphroditic gastropods: conceptual and empirical approaches. Canadian Journal of Zoology.

[bib115] Nikitin ES, Vavoulis DV, Kemenes I, Marra V, Pirger Z, Michel M, Feng J, O'Shea M, Benjamin PR, Kemenes G (2008). Persistent sodium current is a nonsynaptic substrate for long-term associative memory. Current Biology.

[bib116] Núñez PE, Adema CM, de Jong-Brink M (1994). Modulation of the bacterial clearance activity of haemocytes from the freshwater mollusc, *Lymnaea stagnalis*, by the avian schistosome, *Trichobilharzia ocellata*. Parasitology.

[bib117] OECD (2016). Test No. 243: Lymnaea Stagnalis Reproduction Test, OECD Guidelines for The Testing of Chemicals.

[bib118] Oliverio M, Digilio MC, Versacci P, Dallapiccola B, Marino B (2010). Shells and heart: are human laterality and chirality of snails controlled by the same maternal genes?. American Journal of Medical Genetics Part A.

[bib119] Palmer AR (2009). Animal asymmetry. Current Biology.

[bib120] Palmer AR (2016). What determines direction of asymmetry: genes, environment or chance?. Philosophical Transactions of the Royal Society B: Biological Sciences.

[bib121] Park JH, Straub VA, O'Shea M (1998). Anterograde signaling by nitric oxide: characterization and in vitro reconstitution of an identified nitrergic synapse. The Journal of Neuroscience.

[bib122] Patel BA, Arundell M, Allen MC, Gard P, O'Hare D, Parker K, Yeoman MS (2006). Changes in the properties of the modulatory cerebral giant cells contribute to aging in the feeding system of *Lymnaea*. Neurobiology of Aging.

[bib123] Pélissié B, Jarne P, David P P (2012). Sexual selection without sexual dimorphism: bateman gradients in a simultaneous hermaphrodite. Evolution.

[bib124] Perry SJ, Straub VA, Kemenes G, Santama N, Worster BM, Burke JF, Benjamin PR (1998). Neural modulation of gut motility by myomodulin peptides and acetylcholine in the snail *Lymnaea*. Journal of Neurophysiology.

[bib125] Perry SJ, Dobbins AC, Schofield MG, Piper MR, Benjamin PR (1999). Small cardioactive peptide gene: structure, expression and mass spectrometric analysis reveals a complex pattern of co-transmitters in a snail feeding neuron. The European Journal of Neuroscience.

[bib126] Perry KJ, Henry JQ (2015). CRISPR/Cas9-mediated genome modification in the mollusc, *Crepidula fornicata*. Genesis.

[bib127] Phifer-Rixey M, Nachman MW (2015). Insights into mammalian biology from the wild house mouse *Mus musculus*. eLife.

[bib128] Pirger Z, László Z, Kemenes I, Tóth G, Reglodi D, Kemenes G (2010). A homolog of the vertebrate pituitary adenylate cyclase-activating polypeptide is both necessary and instructive for the rapid formation of associative memory in an invertebrate. Journal of Neuroscience.

[bib129] Pirger Z, Crossley M, László Z, Naskar S, Kemenes G, O'Shea M, Benjamin PR, Kemenes I (2014a). Interneuronal mechanism for Tinbergen's Hierarchical Model of Behavioral Choice. Current Biology.

[bib130] Pirger Z, Naskar S, László Z, Kemenes G, Reglődi D, Kemenes I (2014b). Reversal of age-related learning deficiency by the vertebrate PACAP and IGF-1 in a novel invertebrate model of aging: the pond snail (*Lymnaea stagnalis*). The Journals of Gerontology Series A: Biological Sciences and Medical Sciences.

[bib131] Pirger Z, Zrinyi Z, Maasz G, Molnar E, Kiss T (2018). Pond snail reproduction as model in the environmental risk assesment: reality and doubts. Intech Open.

[bib132] Puurtinen M, Emily Knott K, Suonpää S, Nissinen K, Kaitala V (2007). Predominance of outcrossing in *Lymnaea stagnalis* despite low apparent fitness costs of self-fertilization. Journal of Evolutionary Biology.

[bib133] Pyatt FB, Pyatt AJ, Pentreath VW (1997). Short Communication—Distribution of metals and accumulation of lead by different tissues in the freshwater *snail Lymnaea stagnalis* (L.). Environmental Toxicology and Chemistry.

[bib134] Ribeiro MJ, Serfozo Z, Papp A, Kemenes I, O'Shea M, Yin JC, Benjamin PR, Kemenes G (2003). Cyclic AMP response element-binding (CREB)-like proteins in a molluscan brain: cellular localization and learning-induced phosphorylation. European Journal of Neuroscience.

[bib135] Rivi V, Benatti C, Colliva C, Radighieri G, Brunello N, Tascedda F, Blom JMC (2020). *Lymnaea stagnalis* as model for translational neuroscience research: from pond to bench. Neuroscience & Biobehavioral Reviews.

[bib136] Sadamoto H, Hatakeyama D, Kojima S, Fujito Y, Ito E (1998). Histochemical study on the relation between NO-generative neurons and central circuitry for feeding in the pond snail, *Lymnaea stagnalis*. Neuroscience Research.

[bib137] Sadamoto H, Sato H, Kobayashi S, Murakami J, Aonuma H, Ando H, Fujito Y, Hamano K, Awaji M, Lukowiak K, Urano A, Ito E (2004). CREB in the pond snail *Lymnaea stagnalis*: cloning, gene expression, and function in identifiable neurons of the central nervous system. Journal of Neurobiology.

[bib138] Sadamoto H, Kitahashi T, Fujito Y, Ito E (2010). Learning-Dependent gene expression of CREB1 isoforms in the molluscan brain. Frontiers in Behavioral Neuroscience.

[bib139] Sadamoto H, Takahashi H, Okada T, Kenmoku H, Toyota M, Asakawa Y (2012). De novo sequencing and transcriptome analysis of the central nervous system of mollusc *Lymnaea stagnalis* by deep RNA sequencing. PLOS ONE.

[bib140] Samu D, Marra V, Kemenes I, Crossley M, Kemenes G, Staras K, Nowotny T (2012). Single electrode dynamic clamp with StdpC. Journal of Neuroscience Methods.

[bib141] Santama N, Li KW, Bright KE, Yeoman M, Geraerts WP, Benjamin PR, Burke JF (1993). Processing of the FMRFamide precursor protein in the snail *Lymnaea stagnalis*: characterization and neuronal localization of a novel peptide, 'SEEPLY'. European Journal of Neuroscience.

[bib142] Santama N, Benjamin PR, Burke JF (1995a). Alternative RNA splicing generates diversity of neuropeptide expression in the brain of the snail Lymnaea: in situ analysis of mutually exclusive transcripts of the FMRFamide gene. European Journal of Neuroscience.

[bib143] Santama N, Wheeler CH, Skingsley DR, Yeoman MS, Bright K, Kaye I, Burke JF, Benjamin PR (1995b). Identification, distribution and physiological activity of three novel neuropeptides of *Lymnaea*: eflrlamide and pQFYRlamide encoded by the FMRFamide gene, and a related peptide. European Journal of Neuroscience.

[bib144] Santama N, Benjamin PR (2000). Gene expression and function of FMRFamide-related neuropeptides in the snail *Lymnaea*. Microscopy Research and Technique.

[bib145] Schacher S, Castellucci VF, Kandel ER (1988). cAMP evokes long-term facilitation in *Aplysia* sensory neurons that requires new protein synthesis. Science.

[bib146] Scott AP (2012). Do mollusks use vertebrate sex steroids as reproductive hormones? part I: critical appraisal of the evidence for the presence, biosynthesis and uptake of steroids. Steroids.

[bib147] Scutt G, Allen M, Kemenes G, Yeoman M (2015). A switch in the mode of the sodium/calcium exchanger underlies an age-related increase in the slow afterhyperpolarization. Neurobiology of Aging.

[bib148] Skála V, Walker AJ, Horák P (2020). Snail defence responses to parasite infection: the *Lymnaea stagnalis-Trichobilharzia szidati* model. Developmental & Comparative Immunology.

[bib149] Smit AB, Geraerts PM, Meester I, van Heerikhuizen H, Joosse J (1991). Characterization of a cDNA clone encoding molluscan insulin-related peptide II of *Lymnaea stagnalis*. European Journal of Biochemistry.

[bib150] Smit AB, Thijsen SF, Geraerts WP, Meester I, van Heerikhuizen H, Joosse J (1992). Characterization of a cDNA clone encoding molluscan insulin-related peptide V of *Lymnaea stagnalis*. Molecular Brain Research.

[bib151] Smit AB, Thijsen SF, Geraerts WP (1993a). Cdna cloning of the sodium-influx-stimulating peptide in the mollusc, *Lymnaea stagnalis*. European Journal of Biochemistry.

[bib152] Smit AB, van Marle A, van Elk R, Bogerd J, van Heerikhuizen H, Geraerts WP (1993b). Evolutionary conservation of the insulin gene structure in invertebrates: cloning of the gene encoding molluscan insulin-related peptide III from *Lymnaea stagnalis*. Journal of Molecular Endocrinology.

[bib153] Smit AB, Spijker S, Van Minnen J, Burke JF, De Winter F, Van Elk R, Geraerts WP (1996). Expression and characterization of molluscan insulin-related peptide VII from the mollusc *Lymnaea stagnalis*. Neuroscience.

[bib154] Smit AB, van Kesteren RE, Li KW, Van Minnen J, Spijker S, Van Heerikhuizen H, Geraerts WP (1998). Towards understanding the role of insulin in the brain: lessons from insulin-related signaling systems in the invertebrate brain. Progress in Neurobiology.

[bib155] Stephenson R, Lewis V (2011). Behavioural evidence for a sleep-like quiescent state in a pulmonate mollusc, *Lymnaea stagnalis* (Linnaeus). Journal of Experimental Biology.

[bib156] Stewart MJ, Wang T, Koene JM, Storey KB, Cummins SF (2016). A "Love" Dart Allohormone Identified in the Mucous Glands of Hermaphroditic Land Snails. Journal of Biological Chemistry.

[bib157] Stoiber T, Croteau MN, Römer I, Tejamaya M, Lead JR, Luoma SN (2015). Influence of hardness on the bioavailability of silver to a freshwater snail after waterborne exposure to silver nitrate and silver nanoparticles. Nanotoxicology.

[bib158] Swart EM, Davison A, Ellers J, Filangieri RR, Jackson DJ, Mariën J, van der Ouderaa IBC, Roelofs D, Koene JM (2019). Temporal expression profile of an accessory-gland protein that is transferred via the seminal fluid of the simultaneous hermaphrodite *Lymnaea stagnalis*. Journal of Molluscan Studies.

[bib159] Syed NI, Bulloch AG, Lukowiak K (1990). In vitro reconstruction of the respiratory central pattern generator of the mollusk *Lymnaea*. Science.

[bib160] Syed NI, Winlow W (1991). Coordination of locomotor and cardiorespiratory networks of *Lymnaea stagnalis* by a pair of identified interneurones. The Journal of Experimental Biology.

[bib161] Ter Maat A, Pieneman AW, Koene JM (2012). The effect of light on induced egg laying in the simultaneous hermaphrodite *Lymnaea stagnalis*. Journal of Molluscan Studies.

[bib162] Teskey ML, Lukowiak KS, Riaz H, Dalesman S, Lukowiak K (2012). What's hot: the enhancing effects of thermal stress on long-term memory formation in *Lymnaea stagnalis*. Journal of Experimental Biology.

[bib163] Tufi S, Stel JM, de Boer J, Lamoree MH, Leonards PE (2015). Metabolomics to explore Imidacloprid-Induced toxicity in the central nervous system of the freshwater snail *Lymnaea stagnalis*. Environmental Science & Technology.

[bib164] Van Duivenboden YA, Maat AT (1985). Masculinity and receptivity in the hermaphrodite pond snail, *Lymnaea stagnalis*. Animal Behaviour.

[bib165] Van Kesteren RE, Smit AB, De Lange RP, Kits KS, Van Golen FA, Van Der Schors RC, De With ND, Burke JF, Geraerts WP (1995). Structural and functional evolution of the vasopressin/oxytocin superfamily: vasopressin-related conopressin is the only member present in *Lymnaea*, and is involved in the control of sexual behavior. The Journal of Neuroscience.

[bib166] van Kesteren RE, Carter C, Dissel HM, van Minnen J, Gouwenberg Y, Syed NI, Spencer GE, Smit AB (2006). Local synthesis of actin-binding protein beta-thymosin regulates neurite outgrowth. Journal of Neuroscience.

[bib167] Vehovszky A, Szabó H, Hiripi L, Elliott CJ, Hernádi L (2007). Behavioural and neural deficits induced by rotenone in the pond snail *Lymnaea stagnalis*. A possible model for parkinson's disease in an invertebrate. European Journal of Neuroscience.

[bib168] Vehovszky Á, Farkas A, Ács A, Stoliar O, Székács A, Mörtl M, Győri J (2015). Neonicotinoid insecticides inhibit cholinergic neurotransmission in a molluscan (*Lymnaea stagnalis*) nervous system. Aquatic Toxicology.

[bib169] Vigil FA, Giese KP (2018). Calcium/calmodulin-dependent kinase II and memory destabilization: a new role in memory maintenance. Journal of Neurochemistry.

[bib170] Vlaeminck K, Viaene KPJ, Van Sprang P, Baken S, De Schamphelaere KAC (2019). The use of mechanistic population models in metal risk assessment: combined effects of copper and food source on *Lymnaea stagnalis* populations. Environmental Toxicology and Chemistry.

[bib171] Vorontsova YL, Slepneva IA, Yurlova NI, Ponomareva NM, Glupov VV (2019). The effect of trematode infection on the markers of oxidative stress in the offspring of the freshwater snail *Lymnaea stagnalis*. Parasitology Research.

[bib172] Vreugdenhil E, Jackson JF, Bouwmeester T, Smit AB, Van Minnen J, Van Heerikhuizen H, Klootwijk J, Joosse J (1988). Isolation, characterization, and evolutionary aspects of a cDNA clone encoding multiple neuropeptides involved in the stereotyped egg-laying behavior of the freshwater snail *Lymnaea stagnalis*. The Journal of Neuroscience.

[bib173] Wachtel H, Kandel ER (1967). A direct synaptic connection mediating both excitation and inhibition. Science.

[bib174] Wagatsuma A, Sadamoto H, Kitahashi T, Lukowiak K, Urano A, Ito E (2005). Determination of the exact copy numbers of particular mRNAs in a single cell by quantitative real-time RT-PCR. Journal of Experimental Biology.

[bib175] Wan H, Mackay B, Iqbal H, Naskar S, Kemenes G (2010). Delayed intrinsic activation of an NMDA-independent CaM-kinase II in a critical time window is necessary for late consolidation of an associative memory. Journal of Neuroscience.

[bib176] Worster BM, Yeoman MS, Benjamin PR (1998). Matrix-assisted laser desorption/ionization time of flight mass spectrometric analysis of the pattern of peptide expression in single neurons resulting from alternative mRNA splicing of the FMRFamide gene. European Journal of Neuroscience.

[bib177] Yeoman MS, Patel BA, Arundell M, Parker K, O'Hare D (2008). Synapse-specific changes in serotonin signalling contribute to age-related changes in the feeding behaviour of the pond snail, *Lymnaea*. Journal of Neurochemistry.

[bib178] Yeoman MS, Faragher RG (2001). Ageing and the nervous system: insights from studies on invertebrates. Biogerontology.

[bib179] Zelck UE, Janje B, Schneider O (2005). Superoxide dismutase expression and H2O2 production by hemocytes of the trematode intermediate host *Lymnaea stagnalis* (Gastropoda). Developmental & Comparative Immunology.

[bib180] Zhang P, Blonk BA, van den Berg RF, Bakker ES (2018a). The effect of temperature on herbivory by the omnivorous ectotherm snail *Lymnaea stagnalis*. Hydrobiologia.

[bib181] Zhang L, Khattar N, Kemenes I, Kemenes G, Zrinyi Z, Pirger Z, Vertes A (2018b). Subcellular peptide localization in single identified neurons by capillary microsampling mass spectrometry. Scientific Reports.

[bib182] Zimmer EI, Ducrot V, Jager T, Koene J, Lagadic L, Kooijman SALM (2014). Metabolic acceleration in the pond snail *Lymnaea stagnalis*?. Journal of Sea Research.

[bib183] Zonneveld C, Kooijman SALM (1989). Application of a dynamic energy budget model to Lymnaea stagnalis (L.). Functional Ecology.

[bib184] Zrinyi Z, Maasz G, Zhang L, Vertes A, Lovas S, Kiss T, Elekes K, Pirger Z (2017). Effect of progesterone and its synthetic analogs on reproduction and embryonic development of a freshwater invertebrate model. Aquatic Toxicology.

